# Retinal Alterations Predict Early Prodromal Signs of Neurodegenerative Disease

**DOI:** 10.3390/ijms25031689

**Published:** 2024-01-30

**Authors:** Fabio Casciano, Enrico Zauli, Claudio Celeghini, Lorenzo Caruso, Arianna Gonelli, Giorgio Zauli, Angela Pignatelli

**Affiliations:** 1 Department of Translational Medicine and LTTA Centre, University of Ferrara, 44121 Ferrara, Italy; 2 Department of Translational Medicine, University of Ferrara, 44121 Ferrara, Italy; 3 Department of Environment and Prevention Sciences, University of Ferrara, 44121 Ferrara, Italy; 4 Research Department, King Khaled Eye Specialistic Hospital, Riyadh 12329, Saudi Arabia; 5 Department of Neuroscience and Rehabilitation, University of Ferrara, 44124 Ferrara, Italy

**Keywords:** visual impairment, Parkinson’s disease, Alzheimer’s disease, visual acuity, dementia, dopamine, amyloid plaques, neurofibrillary tangles, early detection, prodromal symptoms, retinal degeneration, RGC

## Abstract

Neurodegenerative diseases are an increasingly common group of diseases that occur late in life with a significant impact on personal, family, and economic life. Among these, Alzheimer’s disease (AD) and Parkinson’s disease (PD) are the major disorders that lead to mild to severe cognitive and physical impairment and dementia. Interestingly, those diseases may show onset of prodromal symptoms early after middle age. Commonly, the evaluation of these neurodegenerative diseases is based on the detection of biomarkers, where functional and structural magnetic resonance imaging (MRI) have shown a central role in revealing early or prodromal phases, although it can be expensive, time-consuming, and not always available. The aforementioned diseases have a common impact on the visual system due to the pathophysiological mechanisms shared between the eye and the brain. In Parkinson’s disease, α-synuclein deposition in the retinal cells, as well as in dopaminergic neurons of the substantia nigra, alters the visual cortex and retinal function, resulting in modifications to the visual field. Similarly, the visual cortex is modified by the neurofibrillary tangles and neuritic amyloid β plaques typically seen in the Alzheimer’s disease brain, and this may reflect the accumulation of these biomarkers in the retina during the early stages of the disease, as seen in postmortem retinas of AD patients. In this light, the ophthalmic evaluation of retinal neurodegeneration could become a cost-effective method for the early diagnosis of those diseases, overcoming the limitations of functional and structural imaging of the deep brain. This analysis is commonly used in ophthalmic practice, and interest in it has risen in recent years. This review will discuss the relationship between Alzheimer’s disease and Parkinson’s disease with retinal degeneration, highlighting how retinal analysis may represent a noninvasive and straightforward method for the early diagnosis of these neurodegenerative diseases.

## 1. Introduction

Since ancient times, the eyes have been considered a crucial organ of our body. Indeed, how can we forget the common saying “The eyes are the windows to the soul”? This saying highlights that the eyes often reflect our emotions, fears, and deepest emotional nuances, which the body originates in our brain, suggesting that examining the eyes could hold clinical significance.

The retina is a neural sensory structure that works as an appendage or extension of the central nervous system (CNS), with which it shares several features. In each eye, axonal projection of retinal ganglion cells emerges from the eye via the optic nerves, converges at the ventral diencephalon to form the optic chiasm, and enters the brain. Consequently, the optic nerve and the retina are considered parts of the CNS rather than peripheral sensory organs. Furthermore, the retina and brain share a common embryonic origin. During the early stages of embryonic development, the neural tube evaginates from the anterior neural tube, which gives rise to the forebrain, forming the progenitor of the optic stalk, retina, and retinal pigment epithelium (RPE) [1].

Remarkably, the histology of the retina exhibits characteristics similar to some parts of the brain. Retinal ganglion cells, for instance, exhibit similarities in morphology, neurotransmitters, and signal conditioning circuits to CNS cells and the spinal cord [2].

The name “retina” derives from the well-organized anatomical structure of blood vessels that supply the neuronal part of the retina, forming a layered network containing five classes of neurons responsible for vision [3]. Due to its highly ordered and layered structure, the retina was the first neural structure examined by modern electrophysiologists, enabling the identification of the mechanism underlying signal transmission in the CNS.

The visual system is a complex network of pathways connecting the eyes to the visual cortex of the brain, enabling us to perceive and interpret the world around us. In humans, vision is the most critical sensory system, allowing us to continuously evaluate the surrounding world and objects far from our body.

### 1.1. Image Formation in the Eye

During image formation, the photoreceptors serve as neurocellular units, absorbing photons (units of visible light) and generating electrical signals that enable an optical image to be encoded into a neural image. Vision commences with light entering the eye, which is a form of electromagnetic energy that interacts with the photoreceptors of the retina through a phototransduction mechanism [4]. This initiates a process that generates neural impulses, which subsequently reach the dedicated central brain area. Here, the signals originating from the retina undergo further processing up to conscious visual perception. The light first hits the cornea, allowing the light to enter the eye and focus on the crystalline lens, a transparent biconvex structure located behind the iris, which inverts the image on the retina. The lens’s focus is subtly controlled by the ciliary muscles, which adjust its position to alter the refractive power, enabling it to focus on objects at varying distances. The bent light is then projected onto the retina, where its photoreceptors, the cones and rods, absorb the light and convert it into electrical signals that are transmitted to the brain via the optic nerve.

### 1.2. Neural Organization of the Retina

The retina covers the posterior surface of the eyeball and is a thin and delicate tissue that is approximately 100–300 µm thick. Toward the inner part of the eye, the retina meets the vitreous humor, a transparent substance that fills the eyeball. On the external side, there is initially the retinal pigment epithelium (RPE), which shares the same embryonic origin as the retina proper, and then the choroid, which is a highly vascularized membrane that forms part of the eye’s fibrous tunic [5].

Furthermore, retinal tissue can be considered a neurovascular system through the interaction among neurons, vascular tissue, and glial cells. The retina has two major overlapping layers: the outermost RPE and the innermost neurosensory retina, which lies adjacent to the vitreous body. The neurosensory retina is safeguarded from light by its close relationship with the RPE, which absorbs excess light, as well as having trophic and metabolic roles. The outermost layer of the retina is fed from the choroid, the highly vascularized layer of the eye that supplies the eye with nutrients and oxygen [6].

The neural portion of the retina comprises several types of highly stratified cells interconnected by synapses between their axons and dendrites. Beginning from the scleral side, the signals from photoreceptors are processed and relayed through three layers of neurons that can be distinguished as described next. Initially, the layer of photoreceptors includes cones and rods; then, there is the intermediate layer of bipolar and horizontal cells. Bipolar cells receive the majority of the input that arises from the photoreceptors and directly transmit to the retinal ganglion cells (RGCs). However, horizontal cells are needed to collect signals between multiple photoreceptors and bipolar cells [7]. Finally, the innermost cellular layer includes amacrine and ganglion cells. Amacrine cells route the signals received from bipolar cells and relay them to RGCs [3,8,9]. Noteworthy, some amacrine interneurons, by their release of dopamine (DA), facilitate the transition from dim to bright ambient light conditions, modulating retinal signaling [10,11,12,13]. RGCs, located in the innermost layer of the eye near the vitreous body, project their unmyelinated axons out of the retina in the nerve fiber layer (RNFL), forming the optic nerve [14]. This nerve exits the eye through the sclera at the optic nerve head, also known as the optic disc. Posterior to the globe is the optic nerve, which is covered with myelin produced by oligodendrocytes, similar to other fiber tracts in the CNS.

**Figure 1:**
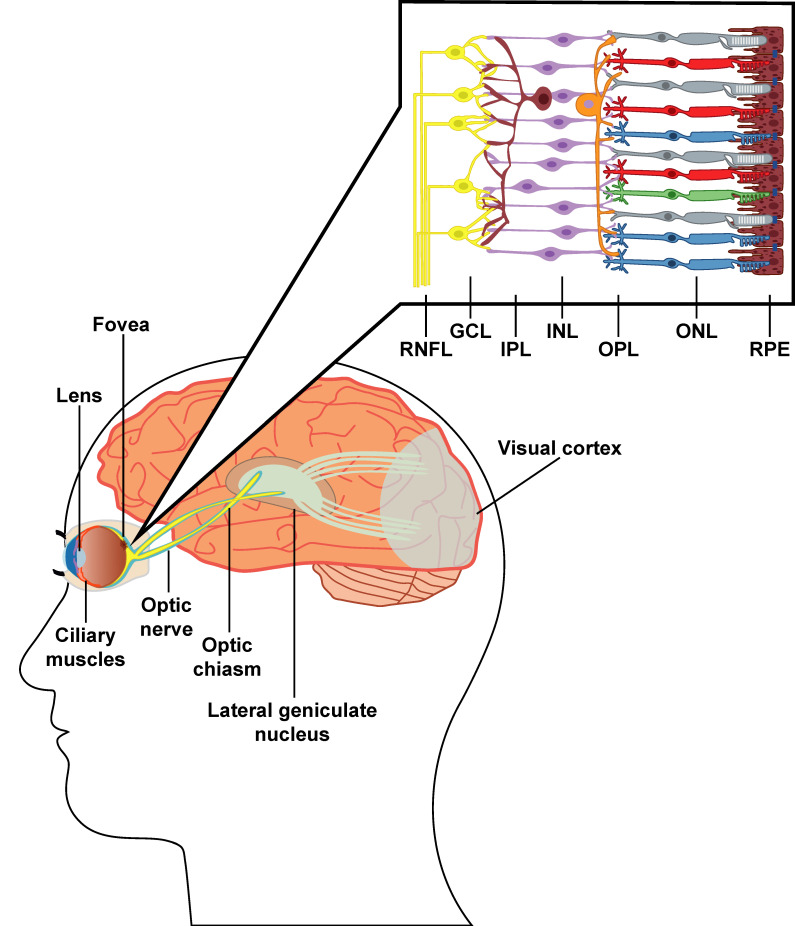
Schematic representation of the visual pathway and retinal layers: RNFL, retinal nerve fiber layer; GCL, ganglion cell layer; IPL, inner plexiform layer; OPL, outer plexiform layer, ONL, outer nuclear layer; RPE, retinal pigment epithelium.

Ganglion cells represent the output elements of the retina, and their axons form the optic nerve fibers that carry visual messages to the brain’s visual centers. As shown in Figure 1, the optic nerve from each eye enters the brain and meets at the optic chiasm; half of the axons from each eye cross to the opposite hemisphere, joining the half of the optic nerve that did not cross. These fiber bundles form the optic tracts, which contain fibers from both eyes. From the optic chiasm, the optic tracts carry information to the lateral geniculate nucleus of the thalamus through optic radiation and then to the primary visual area of the cortex, which receives signals from the contralateral visual field of each eye [15].

The visual cortex is composed of distinct areas, each of which is specialized for the processing of different aspects of the visible world, as relayed from the retinas, such as shape, color, spatial frequency, movement, image edge, visual contrast, spatial orientation, and distance [16,17,18].

### 1.3. The Area of Maximal Visual Acuity

At the center of the retina, about 15 degrees away from the optic disc in visual angle, there is a small, light-sensitive region called the macula (or macula lutea), which is responsible for sharp and detailed vision. Compared with the peripheral retina, the macular retina exhibits distinct characteristics. In this region, the highest density of photoreceptors (specifically, cones) is observed. Furthermore, within the central portion of the macula, a slight depression emerges, which represents the zone of optimal visual acuity. This portion is the fovea. In the foveal region, the concentration of cones reaches its peak, while rods are completely absent. Due to the maximum density of photoreceptors (mainly cones) and the organization of neural connections, the macula is the most sensitive area for discerning fine details compared with the rest of the retina, enabling precise (point) vision. The macula is responsible for central vision, allowing us to focus our gaze on the center of the visual field, where the greatest concentration of light rays converges. The point vision associated with the macular region area allows us to read, recognize a face, see road signs while driving, and distinguish fine details and very small objects, as well as color perception [19,20].

The macula is an extremely delicate region and, consequently, it is particularly susceptible to pathological and degenerative phenomena. Diseases that affect the macula have an immediate negative effect on visual function such as alterations in central visual acuity, with preservation of peripheral vision; reduced contrast sensitivity; changes in the perception of colors, with color appearing dull; and distortion of images [21,22,23,24].

### 1.4. Retinal Electrophysiology Technique

The aim of visual electrophysiology is to assess the electrical events that happen in the visual pathway, evaluating the light signal transduction and electrical transmission from the cellular network of the retina to the visual cortex [25,26]. Noteworthy, these electrophysiological tests should be used as a compendium during a comprehensive clinical investigation along with other ocular imaging tests.

Here, we summarize some of the main electrophysiological tests commonly used in clinical practice that we will discuss in this review.

The electroretinogram (ERG) is a non-invasive diagnostic test that measures the electrical activity generated by retinal neurons in response to light stimuli. These stimuli can be delivered in the form of bright flashes, flickering patterns, and patterns of black and white squares on a checkerboard. ERGs are recorded using a thin fiber electrode placed in contact with the cornea or an electrode embedded within a corneal contact lens, and in this way, they record the electrical activity generated by the retina on the corneal surface. ERG recording has been standardized by the International Society for Clinical Electrophysiology of Vision (ISCEV) to assist ophthalmologists in clinical practice.

Visual evoked potential (VEP) is a diagnostic test that measures the electrical signal that is generated in the visual cortex in response to visual stimulation. By following the standard protocols of the ISCEV, the first portion of the visual pathway starting from the retina, optic nerve, and optic chiasm can be evaluated. The analysis of VEPs is able to give indications of the integrity of these neural portions [27,28].

The electroretinogram (PERG) is an electrophysiological ophthalmological test that provides a quantitative, non-invasive measurement of central retinal function by recording the electrical activity of macular and retinal ganglion cells. It can help distinguish between diseases of macular nerve dysfunction and those of the optic nerve. It is performed using conjunctival and skin electrodes using a visual stimulus consisting of a chessboard in which black and white elements alternate with regular frequency. It can be an indicator of dysfunctions of the macular region and can be used in the diagnosis and monitoring of pathologies that determine a primary impairment of the retinal ganglion cells or visual deficits affecting the optic nerve [29].

## 2. Why Investigate the Prodromal Signs of Neurodegenerative Diseases in the Retina?

In light of the existing anatomical similarities between the retina and the brain, it seems natural that ocular modifications could be used as indicators of underlying brain pathologies. Indeed, ophthalmological analyses have revealed changes in the eyes and vision of patients with disorders of the nervous system, including stroke, multiple sclerosis, Parkinson’s disease (PD), and Alzheimer’s disease (AD). In many of these diseases, ocular manifestations precede the emergence of central symptoms, suggesting that ophthalmological investigations may provide a noninvasive tool for early diagnosis [30,31,32].

Additionally, microvascular modifications in the retina, such as arteriovenous nicking, retinal hemorrhage, and arteriolar narrowing, may be linked with an increased risk of white matter lesions in the brain, possibly arising from cerebral ischemia, stroke, and stroke-related mortality and, therefore, could be considered prodromal signs [33,34,35,36,37].

Patients with PD frequently exhibit early-onset visual impairment that mirrors retinal alterations and electrophysiological modifications. Intriguingly, questionnaire studies have revealed that up to 70% of PD patients report visual symptoms. These impairments entail a decrease in visual acuity, reduced contrast sensitivity, aberrant spatial contrast sensitivity, and color discrimination, often associated with difficulties in performing complex visual tasks [38,39,40].

Similarly, vision impairments are common among patients with dementia, including those with AD and Lewy body dementia (LBD) [41]. Interestingly, a cohort study of 1061 elderly individuals revealed that visual impairment was associated with an increase in incident dementia [42]. The visual deficit in AD may exhibit a dual etiology, as it may result either from changes in brain regions associated with the visual system or from retinal degeneration that drives the loss of low-level visual function [43].

Given the complex interplay between the eye and the brain, retinal screening could become a useful valuable window in which to view neurodegenerative diseases. In this review, we will explore the two most prevalent neurodegenerative diseases, i.e., Alzheimer’s disease and Parkinson’s disease, in which the retina can highlight prodromal signs of neurodegeneration. Table 1 summarizes the main references used in this review related to visual dysfunctions in Parkinson’s disease and Alzheimer’s disease.

## 3. Alzheimer’s Disease

The name Alzheimer’s disease was coined by Dr. Emil Kraepelin in honor of his student, Alois Alzheimer, a clinical psychiatrist and neuroanatomist who found plaques and neurofibrillary tangles in the brain histology of a 50-year-old woman, with presenile dementia, who died in 1906. Alzheimer’s disease is the most common form of dementia, amounting to at least 60–80% of the cases in patients ages 65 and older in the USA [112,113]. It has been estimated that the number of AD patients will grow from 6.5 to 13.8 million Americans by 2060 [112,114]. The symptoms of AD are characterized by progressive loss of memory, language, executive and visuospatial abilities, and overall cognitive decline associated with neurodegeneration that disrupts daily life.

The pathogenesis of AD involves the degeneration of neurons and altered neuronal connection caused by aggregation of proteins in the basal forebrain and cerebral cortex [115,116].

### 3.1. AD Biomarkers

Technological advancement along with the advent of biomarkers enables improved clinical diagnosis of AD. For instance, the National Institute on Aging and the Alzheimer’s Association defined these biomarkers as the framework of the A/T/N (amyloid/tau/neurodegeneration) system for Alzheimer’s disease diagnosis.

The AD hallmarks include neurodegeneration, the deposition of amyloid β (Aβ) plaques, the intracellular accumulation of neurofibrillary tangles (NFTs) composed of tau protein, and the apolipoprotein E (APOE) genotype. Comprehensive neuropathological processes can be assessed using blood, plasma, cerebrospinal fluid (CSF), and bioimaging biomarkers. Biofluid tests quantify the Aβ42 to Aβ40 ratio, total tau protein, and phosphorylated tau protein (p-tau), while nuclear medical imaging scans using Aβ and tau positron-emission tomography (PET) imaging biomarkers allow for the assessment of the risk of disease progression [117,118]. Amyloid β aggregates originate from the cleavage of amyloid beta precursor protein (APP) into short Aβ peptides of about 4 kDa [119]. These monomers bind together to form oligomers and insoluble plaques [120]. Aβ40 is the most abundant form of amyloid in the brain, while Aβ42 is the main species involved in forming amyloid plaques. Indeed, a decrease in the CSF Aβ42 to Aβ40 ratio in AD patients reflects the intraparenchymal accumulation of Aβ42 [121]. This observation is confirmed by PET scanning of amyloids, which highlights deposition in various brain regions [122]. Several mechanisms have been suggested to contribute to the neurodegeneration induced by amyloid toxicity, including mitochondrial dysfunction with Ca^2+^ release and oxidative stress [123,124], ion channel pore formation in neuronal membranes [125], loss of hippocampal long-term potentiation with memory and learning impairment [126], neuronal hyperactivation by the suppression of glutamate reuptake [127,128], and receptor binding (e.g., cellular prion protein, receptor for advanced glycation endproducts RAGE, p75 neurotrophin receptor p75NTR, neurexin1 NgR1, ephrin-B2 EphB2, and FcγRIIb receptor) [129].

Although the accumulation of insoluble Aβ is recognized as a factor in the pathogenesis of AD, tau aggregation and neuroinflammation by damaging neuronal cells also play a role in AD development [130]. Phosphorylated tau protein found in CSF at autopsy more strongly correlates with cognitive disease status than Aβ plaques PET scanning [131], confirming that plasma quantification of p-tau181 and p-tau217 are useful to discriminate AD from other key neuropathologies in older adults [132,133]. Tau proteins are essential for the proper formation of microtubules in a phosphorylation-dependent manner. Modification of tau phosphorylation leads to the loss of its function, and hyperphosphorylated tau protein leads to the formation of cytoplasmic NFTs, although it may also be found in the brain extracellular space [134].

The presence of amyloid plaques in the brain has been observed to correlate with increased levels of specific forms of amyloid beta proteins, such as Aβ42, or changes in the ratio of Aβ42 to Aβ40 [117,135,136]. Aβ deposition originates from the sequential proteolytic cleavage of the integral membrane amyloid precursor protein (APP), mediated by β-secretase and γ-secretase [137]. β-secretase produces a soluble APPβ C-terminal fragment (βCTF) also known as C99. C99 is an intermediate that will go to further cleavage by γ-secretase, releasing two amyloid β amino peptides, Aβ40 and Aβ42 [137]. Although Aβ40 is the main form accumulated in the cerebral spinal fluids, the principal component of amyloid plaques in the AD brain is Aβ42 [138,139]. Indeed, in the brain of AD patients, the Aβ40 concentration decreases from mid-20, suggesting an age-dependent metabolism of soluble Aβs [140]. In vitro studies show that Aβ42 and Aβ40 form interlaced amyloid fibrils, which may contribute to the preferential deposition of Aβ42 in AD brains [141,142]. Accordingly, pharmacological studies with γ-secretase inhibitor reveal that the turnover of Aβ40 was generally faster than Aβ42 [143]; furthermore, Aβ42/40 ratios can be regulated via differential degradation of Aβ species by Cathepsin D (CatD), where Aβ42 acts as potent inhibitor of CatD itself, thus leading to an increase in the cerebral Aβ42/40 ratio [144].

Therefore, given the relationship between the eye and PD, it appears that understanding the association between PD and visual disorders could be beneficial for predicting disease development and progression.

### 3.2. AD Etiology

AD disease has a complex, multi-factor etiology, resulting from the interplay of genetic and environmental factors. Genetically, AD susceptibility is influenced by a broad spectrum of genes, mainly grouped into risk genes and deterministic genes. The former genes increase the odds of developing the disease, while the latter genes directly cause the disease. Over 100 risk genes are suspected to contribute to the disease, and among these, the apolipoprotein E (APO-E) gene, particularly the APOE-Ɛ4 allele, is the most strongly associated with AD risk [145,146,147]. Therefore, to assess genetic risk, it may be helpful to search Genetic Testing for specific variants of the APOE gene.

A small fraction of individuals, approximately 1–5% between the ages of 30 and 60, with Alzheimer’s disease develop an early-onset form of the disease. These early-onset forms are often attributed to rare mutations in three genes encoding APP and presenilin 1 and 2 (PSEN1/PSEN2) [148,149]. On the environmental front, modifiable risk factors for AD include hypertension, smoking, diabetes, depression, head injury, physical inactivity, depression, low educational attainment, and obesity [150].

### 3.3. Disease Staging of AD

The World Health Organization (WHO) defines Alzheimer’s disease as a progressive neurodegenerative disorder. The diagnosis of Alzheimer’s dementia is assessed with cognitive and memory testing, thinking skills, behavioral evaluations, and a series of tests to exclude other potential causes of cognitive decline. Cognitive and memory tests are used to assess the patient’s ability to remember information, think critically, and solve problems. Brain imaging radiological investigations, such as magnetic resonance imaging (MRI) or positron emission tomography (PET), can provide useful insight to allow for the observation of structural changes or anomalies associated with Alzheimer’s disease. By comparing the results of individual assessments with the diagnostic criteria for Alzheimer’s disease defined by the National Institute on Aging–Alzheimer’s Association (NIA–AA) guidelines, the physician will be able to make a precise diagnosis [117]. These guidelines stratify individuals into three clinical stages according to the presence of cognitive impairment along with one or more alterations of biomarkers (i.e., MRI, PET, and CSF): preclinical (early), with brain changes starting up but without clinical symptoms; mild cognitive impairment, also referred to as the prodromal stage of AD (intermediate), where noticeable memory/thinking problems appear; and Alzheimer’s dementia (late), the advanced stage of the disease that significantly impacts the person’s independence [151,152]. Each clinical stage reflects neuropathologic changes in the brain as defined by AD neuropathological changes, which describe the presence of Aβ and tau protein deposition as well as the density of neuritic plaques, progressing in a predictable manner in the brain [153].

### 3.4. Signs of Visual Dysfunction in Alzheimer’s Disease

The literature suggests that vision impairments are common in AD patients, and they are mainly due to the involvement of the brain regions associated with the visual system [154]. Although visual problems are common in the elderly, people with dementia have a high prevalence of visual problems compared with the general population [155], thus reinforcing the idea of the potential use of visual function analysis in the identification of Alzheimer’s neurodegenerative disease. Among the clinical features of AD, these include defects in lower-order visual functions affecting visual acuity, visual field, saccades and pursuit eye movements, color vision, and contrast sensitivity [43,96,156].

Surprisingly, AD patients, with a high genetic risk of developing Alzheimer’s disease (i.e., carrier of at least one ɛ4 allele for the ApoE gene), have shown an unexpected increase in visual acuity and contrast sensitivity [87].

Furthermore, higher-order visual functions (i.e., cognitive operations with visual information) also appear defective, with reduced visual identification and visuospatial processing [157,158]. Additionally, AD patients may experience visual and somatic (e.g., olfactory, tactile) hallucinations, especially at the advanced stages of the disease [159]. Interestingly, a recent systematic review by Kuźma et al. highlights how visual impairments are an indicator of risk for all-cause dementia [160] and become a potential modifiable risk factor for dementia [161]. These symptoms have been partially associated with the deposition of NFTs and amyloid β aggregates in brain regions (i.e., the visual cortex and the three nuclei of the subcortical area: pulvinar, lateral geniculate, and suprachiasmatic nucleus), and in the eyes, with a retinal degeneration that shows retinal thickness and loss of optic nerve axons projections [70,78,79,80,81,82]. Notably, retinal deposition of Aβ aggregates differs from classical Aβ plaques in the brain with a regional deposition in the retina. Indeed, immunohistochemical analysis of postmortem retinas from AD patients reveals globular plaques consisting of multiple amyloid cores, occasionally associated with pericytes, with a characteristic spatial distribution in mid-peripheral rather than central regions of retina specimens, mainly located around retinal ganglion cells [57,62]. All these observations are consistent with the alteration in the morphology and size of dendritic processes of retinal ganglion cells seen by La Morgia et al. in 2016 [69]. Notably, in 2018, Santos and colleagues observed a myelinated axonal loss in the retinal nerve fiber layer that appears weakly correlated with the integration of audio-visual information, suggesting that these changes may not be the most sensitive early markers of mild cognitive impairment [70]. On the other hand, in vivo studies show that in the visual cortex, completely functionally intact NFT-bearing neurons are able to integrate dendritic inputs and have a stable baseline resting calcium level [162]. Furthermore, studies in AD animal models have shown retinal deposition of NFTs and amyloid β aggregates that precede the accumulation in the brain [63,64], where retinal degeneration may be driven by the dysregulation in retinal autophagy and cell death due to the accumulation of α-synuclein (pSer129) and tau protein [55]. Interestingly, the APP/PS1 mouse model of AD showed markers of immunoreactivity of amyloid β load in the retina of the 27-month mouse, consistent with recruitment of macrophages and atrophy of the RNFL as well as loss of cells in the ganglion cell layer (GCL) [65]. Furthermore, using electrophysiology and histological approaches, APP-PS1 mouse AD models reveal that about 30% of Aβ plaques are localized to the inner nuclear layer (INL) with a reduction in the inner retinal electrophysiological responses associated with marked apoptosis in the inner retina [163]. Overall, these findings indicate that Aβ accumulation may disrupt the retinal physiology at all levels (i.e., RNFL, GCL, IPL, OPL, and INL, as seen in Figure 1) [65,66,67,68].

These results are in line with findings in human postmortem retinas of patients with AD, where the accumulation of amyloid β aggregates may consequently lead to the thickening of the inner and outer layers of the retina [83]. Recently, Koronyo et al. established a curcumin-based method to enumerate retinal Aβ aggregates, finding a 2.1-fold increase when comparing retinas from AD patients to healthy controls [62]. This highlights the retina as an optimal location for future AD in vivo imaging of Aβ aggregates as surrogate markers of brain Aβ levels. This technique can be further enhanced using hyperspectral imaging of the retina, which can predict the brain Aβ load without labeling [164,165], becoming a useful in vivo marker for the early identification of brain Aβ plaques.

Along with Aβ deposition, p-tau deposition may also be another key marker of retinal AD patients. In 2018, den Haan et al. found a consistent topographical pattern of p-tau deposition with a histological diffuse signal for p-tau in the inner and outer plexiform layers of the postmortem retina of AD patients [59]. These results are in line with the results obtained by Harrison et al. in a transgenic mouse model of frontotemporal dementia, where the IPL and INL showed an increase in deposition of p-tau with a further accumulation in axons forming the RNFL [56]. Furthermore, these observations were confirmed by Walkiewicz et al., who compared the retinas of 164 individuals with and without AD, finding that retinal p-tau deposition correlated with age, AD progression, and visual impairment, thus highlighting the presence of primary retinal tauopathy molecular distinct from that of cerebral [61].

Vascular pathology is another key feature of the eye in AD patients, as well as in other ocular vascular diseases (i.e., diabetic retinopathy, retinal and macular edema, retinal vein occlusion), where the vascular damage alters nutrient supply and the Aβ clearance, further promoting retinal degeneration [3,166,167,168,169,170]. Further studies of postmortem retinas have identified the deposition of Aβ aggregates and NFTs in ganglion cells, amacrine cells, horizontal cells, and Müller cells of the inner and outer plexiform layers, which correlate with the cortical cerebral pathology burdens, demonstrating the relationship between ocular–brain pathology and cognitive impairment [57,58,59,60].

### 3.5. Ophthalmological Tools to Study Eyes in AD Patients

Objective assessment of the retina and optic nerve function is commonly achieved using pattern electroretinogram (ERG) and pattern visual evoked potential (VEP) tests. Interestingly, those evaluations highlight that dysfunction of retinal ganglion cells and the optic nerve can be observed during AD and are characteristic of the early stages of the disease [106,108,171]. However, only the RNFL thickness seems to strongly correlate with ERG tracing [107]. Accordingly, Arruda et al. recently suggested a standardized and validated method for the use of flash visual evoked potential-P2 (FVEP-P2) in the diagnosis of Alzheimer’s dementia [111].

Optical coherence tomography (OCT) analysis is a non-invasive technique commonly used in clinical practice to quickly measure the RNFL and optic nerve thickness. It has been used successfully in patients with AD by performing comparative analyses of retinas from healthy patients and AD patients. According to the results obtained from postmortem retinas, the comparative analysis shows the thinning of the ganglion cell complex (GCC), a three-retinal-layer structure composed of the nerve fiber layer, ganglion cell layer, and the inner plexiform layer. Interestingly, in AD, this modification appears to mainly involve the inner cell layer and not the outer retinal layers [74]. Notably, in a population-based study involving approximately 3000 patients, Mutlu et al. demonstrated that dementia and AD may be associated with RNFL thinning [75]. Indeed, the retinal thickness appears to be a hallmark of patients with mild cognitive impairment (MCI) and is paralleled with dementia progression toward AD [77,172,173]. However, it is important to note that RNFL thinning may also be a nonspecific indicator of cognitive decline, as shown by Paquet et al., who found no significant retinal thinning when comparing retinas from patients with MCI and mild AD patients [174].

Therefore, based on the findings described above, retinal analysis may have significant additive value in providing clinical signatures for the prediction of AD.

## 4. Parkinson’s Disease

In 1817, Dr. James Parkinson first described the symptoms of a disease affecting physical movement, including bradykinesia, muscle stiffness, tremors, gait difficulties, and postural instability, in his monography “An essay on the shaking palsy” [175]. PD also presents symptoms not related to movement, also called “non-motor symptoms”, including sleep disorders, depression, anxiety, cognitive impairment, constipation, fatigue, and visual dysfunction [176]. These non-motor symptoms may manifest at the early stage of the disease as well as throughout the course of the disease.

Parkinson’s disease is a progressive neurodegenerative disease mainly seen in elderly individuals, with an incidence of 1 to 3% among people older than 65 years. The incidence is higher in males than females and increases with age [177,178].

A hallmark of Parkinson’s disease is the gradual degeneration of dopaminergic neurons and the subsequent decline in dopamine levels within the substantia nigra pars compacta.

### 4.1. The Dopaminergic System in the CNS, Retina, and Olfactory Bulb

Dopamine is the most abundant and studied catecholamine in the vertebrate retina, but many aspects of its function in the retina remain unclear. Dopamine, in addition to its well-established effects as a neuromodulator in the CNS, regulating reward, addiction, motivation, and fine motor control, has been recognized to have important and different functions in sensory systems such as the retina and olfactory bulb [179,180,181]. There, DA is produced endogenously and modulates incoming signals, where it sharpens the sensory processing of visual and olfactory information.

Dopamine’s role in the retina can be indicated as a chemical messenger for light adaptation [182]. The DA control mechanism in the retina allows for transition signaling from dim lights such as night lights to intense daylight ones, while in the olfactory bulb, DA regulates odor discrimination and detection. These modulatory effects of DA are essential both for vision and olfaction. Interestingly, in the retina and olfactory bulb, electrophysiological studies of the two subpopulations of dopaminergic cells show similarities in circuitry, indicating how visual and olfactory systems are similar, even though they are functionally distinct [10].

In the retina, DA is released by a unique set of amacrine cells and activates D1 and D2 DA receptors distributed throughout the retina. Retinal amacrine cells release DA with a circadian rhythm controlled by light exposure, with high DA levels occurring during the day and low levels occurring at night. Because of this light-sensitive variation in DA concentration, it has been postulated that DA plays a role in the transition from a dark- to a light-adapted state [12,13,183].

Dopamine in the retina is also a regulator of high acuity, light-adapted vision [184], and photoreceptor coupling in the retina [185]. The lack of tyrosine hydroxylase (TH) leads to deficits in contrast sensitivity and acuity [51] related to light reduces ganglion cell spike firing via DA receptor activation [13].

The presence of α-synuclein aggregates in the retina, an abnormal protein associated with neurodegenerative diseases, has also been observed in PD patients [52]. In PD, we witness a loss of dopaminergic neurons in the substantia nigra of the brain subsequent to the accumulation of Lewy bodies, which are hallmark pathological structures formed by alpha-synuclein aggregates. In recent years, this protein has been studied as a biomarker, i.e., as a biological indicator that can be related to the onset or development of Parkinson’s disease [54,186].

Interestingly, a study involving 30 patients with idiopathic rapid eye movement sleep behavior disorder (iRBD), a highly predictive early sign of Lewy body disease, also revealed impaired contrast sensitivity. Furthermore, the authors observed thinning of the macular inner retinal layer, a finding that unexpectedly correlated with olfactory and dopaminergic deficits [41].

Visual and olfactory systems, in addition to being affected by DA, exhibit striking similarities in their anatomical structure organized into distinct layers. Electrical signals propagate through a well-defined pattern of neurons in both systems, and both utilize lateral inhibition to sharpen sensory processing [10]. Interestingly, as described for the retina, partial or total olfactory deficits (including hyposmia and anosmia) are also observed for the olfactory system as preclinical stages of PD, preceding motor symptoms by years [187]. This suggests that olfactory dysfunction, revealed with smell tests, may predict PD before the emergence of motor symptoms [188,189,190,191]. In contrast with the retina, DA neuron numbers in the olfactory bulb of people with PD and in Parkinsonian rodent models are dramatically higher [192,193,194]. A possible explanation for this paradox may be attributed to the process of adult neurogenesis that occurs in the olfactory bulb, which would lead to a compensatory mechanism, although the reason for this increase in people with PD remains unclear [195,196,197,198].

### 4.2. PD Biomarkers

Currently, while the complete pathogenesis of PD needs to be fully uncovered, identifying novel biomarkers could furnish valuable insights before clinical symptoms manifest [199]. Therefore, molecular evidence along with motor and imaging biomarkers should be combined to obtain a complete diagnostic panel.

In recent years, in addition to traditional diagnostic methods, the literature has suggested the potential use of blood biomarkers to assess disease-related changes in the brains of patients with Parkinson’s disease. Surprisingly, α-synuclein, which is a key marker of neuronal damage, has been detected in red blood cells, although conflicting data have emerged regarding the correlation between protein levels and PD pathology [200]. Furthermore, there is evidence demonstrating that the presence of Aβ protein in the CSF and blood of PD patients may be associated with cognitive impairment [201,202,203], although evidence shows that the title of Aβ peptides in plasma might derive indirectly from platelets, which could play a role in Aβ transport and deposition in the brain [204]. However, the precise mechanism by which Aβ in the CSF and blood contributes to cognitive decline in PD remains to be fully elucidated.

Neurofilament light chain (NfL) is also a valuable biomarker for the diagnosis of CNS disorders, including multiple sclerosis [205]. Recent studies have further shown that NfL is a promising biomarker for differentiating between idiopathic PD and atypical Parkinsonian syndromes [206]. Notably, Diekamper et al. found a correlation between Nfl levels of the CSF and the severity of nigrostriatal degeneration in PD patients, reflecting striatal dopamine deficiency following nigrostriatal degeneration [207].

### 4.3. PD Etiology

The pathogenesis of PD hinges on the loss of dopaminergic neurons in the substantia nigra pars compacta (SNpc), accounting for up to 70% of the nervous tissues by the time of death. This loss is accompanied by concomitant denervation of their axons that project to the caudate nuclei and putamen of the striatum along the nigrostriatal pathway. The loss of dopaminergic neurons is subsequent to the intracellular accumulations of α-synuclein (α-syn) misfolded protein, leading to the formation of Lewy bodies in SNpc [208] with associated dementia. In healthy cells, α-syn plays a role in the clustering of synaptic vesicles to maintain neurotransmitter release and the reuptake cycle [209,210]. Among other factors contributing to neuronal degeneration are mitochondrial dysfunction, autophagy impairments, and neuroinflammation. Numerous studies highlight that the widespread accumulation of α-synuclein relies on the consequence of impaired autophagic and lysosomal degradations [211].

PD is a complex, multifactorial neurodegenerative disease, where environmental factors (i.e., cigarette smoking, caffeine, pesticide, and heavy metals) interplay with genetic ones [212]. In addition to those factors, PD is also associated with cardiometabolic disturbances, where cholesterol seems to be engaged with a controversial role in PD neuropathology [213]. Genetic research studies have identified about 20 different causative genes that increase the risk of developing the disease. The first evidence of this comes from mutations in SNCA (encoding α-synuclein), which were identified in 1997. Since then, other genes have been identified as heritable components of PD, including mutations in PARKIN, PINK1, LRRK2, and DJ-1 and variants of GBA1. Among these, LRRK2 seems to be a common risk factor for PD, with different distributions in Asian and Caucasian populations based on polymorphic variants of the LRRK2 gene [214,215].

Various findings support the evidence of a relationship between molecular patterns in mitochondria and PD. Initial observations pointed out that mitochondrial toxins trigger Parkinsonian symptoms by inhibiting the respiratory chain [216]; later, genetic studies of PD identified mutations in genes involved in mitochondrial function and degradation. These include autosomal dominant (i.e., SNCA, LRRK2, and VPS35) and recessive transmitted genes (i.e., Parkin, PINK1, and DJ-1). All these genes are in various ways involved in a common pathway for regulating mitochondrial function, mediating mitophagy, and mitochondrial mobility [217]. Furthermore, dopaminergic neurons have a high energy demand, rendering them susceptible to degeneration in SNpc [218]. In this context, the insufficient clearance of α-synuclein impacts mitochondrial function. Notably, α-syn possesses a mitochondrial targeting sequence (MTS) that mediates its mitochondrial membrane (inner and outer) binding as well as its localization in the intermembrane space. This association is linked to α-syn’s maintenance of mitochondrial activity and morphology; disruptions in this association lead to increased Ca^2+^ levels in mitochondria, subsequently causing oxidative stress and cytochrome C release [211,219]. The accumulation of defective mitochondria resulting from the aforementioned autophagic defect further accelerates the disease progression in a vicious cycle.

Tau proteins are a group of proteins that play a physiological role in stabilizing the neuronal cytoskeleton by preventing the dissociation of tubulin [220]. However, they are commonly altered in neurodegenerative diseases, such as Alzheimer’s disease, where tau proteins have a well-established role as a primary biomarker. While Parkinson’s disease was not initially linked to tau protein accumulation, recent studies have revealed findings of tau pathology in PD, with the characteristic of tau aggregate accumulation known as neurofibrillary tangles [221]. Indeed, in Parkinson’s disease, there is a hyperphosphorylation of tau proteins, which may interact with α-syn, leading to a toxic interaction that can further misfold each other, as evidenced by protein deposition and a gain in toxic function that disrupts axonal transport [222,223,224].

Neuroinflammation is more common in people with Parkinson’s disease than in those who do not have the disease, and it plays a significant role in the pathogenesis of Parkinson’s disease compared with other neurodegenerative diseases. Inflammatory processes in PD are driven by the activation of glial cells and astrocytes, with the accumulation of inflammatory cytokines, which is also found in postmortem PD brains. During the disease, dying neurons release damage-associated molecular patterns (DAMPs) such as nucleotides, matrix metalloproteinases, and aggregated proteins, which may trigger purinergic signaling and Toll-like receptors (TLRs) [225]. This, in turn, favors the acquisition of the M1 proinflammatory phenotype by microglia, leading to the release of pro-inflammatory cytokines such as IL-1α, IL-1β, IL-6, and TNF-α, which have been found in the striatum as well as in the nervous system of postmortem samples [226]. Pro-inflammatory cytokines and chemokines also induce an inflammatory phenotype in astrocytes, which amplifies neuronal cell death by compromising the blood–brain barrier (BBB) with the recruitment of lymphatic cells from the systemic circulation [226,227].

### 4.4. Disease Staging of PD

The diagnosis criteria for Parkinson’s disease are based on the “UK Parkinson’s Disease Society Brain Bank Clinical Diagnostic Criteria” (UK PD Brain Bank Criteria), primarily settled on clinical evaluation and assessment of symptoms such as bradykinesia, which is characterized by slowness in initiating voluntary movement, along with a progressive reduction in the speed and amplitude of repetitive actions. In addition, at least one of the following symptoms must be present: muscular rigidity, rest tremor with the frequency of 4–6 Hz, and postural instability not associated with visual, vestibular, cerebellar, or proprioceptive dysfunctions [228].

It is well recognized that PD is characterized by the deposition of α-synuclein in the substantia nigra. However, it is noteworthy that α-synuclein deposition may also affect the entire CNS as well as the autonomic nervous system, which may predict prodromal symptoms of Parkinson’s disease [229,230].

The latent period between the initial lesional neurodegeneration and the onset of PD symptoms can be many years [231]. According to the major symptoms observed in PD patients, the “Movement Disorders Society (MDS) task force” proposed that PD can be categorized into three clinical stages: (i) the preclinical phase; (ii) the premotor phase, also known as prodromal (with non-motor symptoms); and (iii) the motor phase (advanced phase) [176,232,233].

### 4.5. Signs of Visual Dysfunction in Parkinson’s Disease

Given the important role of DA in the retina, it is of great interest to understand how dopaminergic deficiency, which occurs in Parkinson’s disease, might affect retinal functions [30,234].

The first experimental evidence to understand how dopaminergic deficiency occurs in the PD retina showed that there is reduced tyrosine hydroxylase immunoreactivity of dopaminergic cells in patients with Parkinson’s disease. Tyrosine hydroxylase (TH) is the rate-limiting enzyme in the synthesis of DA and is used in immunohistochemistry to identify DA neurons in the retina [46]. Subsequent postmortem studies in PD patients confirmed these data. These studies, together with previous electrophysiological and psychophysical evidence of retinal dysfunction in Parkinson’s disease, led to support the hypothesis that the changes in vision observed in patients with PD are due to a dopaminergic deficiency [47,48,49,50,235].

To verify whether PD patients have changed vision compared with optimal vision, it is advisable to monitor some fundamental visual abilities, such as color vision, contrast sensitivity, visual acuity, and motion perception, and analyze the retinal structure.

To measure visual acuity, a Snellen test with a high-contrast target recognition task can be used. Although impaired visual acuity has been reported in PD patients, there have been limited studies evaluating specifically the visual acuity of the PD population [85,86]. Vision loss may also be associated with other disorders that may occur in PD patients, such as an increased risk of visual hallucinations [236,237], which is also reported in Alzheimer’s patients [238,239]. Diminished visual acuity is a risk factor for visual hallucination, a variety of visual phenomena that can range from simple flashes of light to complex depictions [240,241,242].

In addition, Parkinson’s disease patients demonstrated impairments of visual attention and spatial and motion detection compared with controls [97,98,99].

Using specific clinical tests for color vision, it has been highlighted that deficits have been found in Parkinson’s disease [89,90,91].

Our retina has evolved to signal contrast, enabling us to identify objects under different levels of illumination. The contrast sensitivity test determines how patients can see under high-contrast conditions or under real-world low-contrast conditions. During the test, the patient is asked to detect the lowest level of light frequencies presented in some images. Sinusoidal patterns varying in luminance across a grating pattern are used, and the different size gratings are called spatial frequencies. The luminance of the grating is varied from 0.5% contrast to 90% contrast. Several studies have shown contrast sensitivity loss at a variety of spatial frequencies in one or both eyes of PD patients [93,94,95].

Interestingly, some data showed that contrast sensitivity and color vision improve following L-DOPA administration in PD patients [45,243,244], further suggesting that dopaminergic deficiencies in the retina may be the underlying cause.

Promising markers for the combined diagnosis and prognosis of PD come from visual disorders. Visual disorders, including impairments in eye movements, pupillary function, visual acuity, color vision, contrast sensitivity, and blink reflex, are prevalent symptoms among patients with Parkinson’s disease. Furthermore, changes in blink rate can lead to abnormal tear film and dry eyes that further compromise vision. The rate of these ophthalmological disorders with clinical relevance approaches 92% among individuals with PD, with dry eyes being the most common followed by misalignment of the eye and optic nerve disorders [245]. It is noteworthy that although eye symptoms are common in Parkinson’s disease, they may not only be associated with the disease course but may also be the symptoms of other conditions, such as age-related changes. Proper visual functions have a huge impact on PD patients because they may compensate for the lack of motor function automaticity. Indeed, the loss of vision, combined with postural instability and gait disturbances, can increase the risk of falls and related injuries [246].

Notably, the early stage of PD seems to be characterized by defects in color vision and contrast sensitivity, with a color discrimination deficit worsening with disease severity [88,92].

Interestingly, the visual symptoms may manifest in the early stages of the disease, suggesting that the disease may originate in the eye and progress to the brain. Recently, Acuña et al. demonstrated in a mouse model that intravitreal injection of preformed α-synuclein fibrils leads to their spread into the brains of mice via the visual pathway, thus reinforcing the close association between eye–brain pathology [53].

In the context of the molecular pathophysiology of PD, all these visual symptoms have been partly associated with retinal DA deficiency, as demonstrated by the positive effects of levodopa and apomorphine on improving contrast perception [44,45].

While retinal DA deficiency may be the primary cause of these visual disorders, the orientation-specific impairment due to the cerebral cortex should not be excluded as the loss of dopaminergic neuronal projection [50,247,248,249]. Additionally, DA activates neurons through D1 and D2 DA receptors, which are also expressed throughout the visual cortex, thus opening an interesting avenue for further characterizing PD [250].

It should also be noted that a recent experimental study demonstrated that the data obtained from comparing idiopathic PD patients with control subjects do not support the presence of a difference in VEP amplitude and latency. This finding suggests that retinal analysis may not be a reliable method for monitoring the status of dopaminergic neurodegeneration and axonal loss in PD [251].

### 4.6. Structural Changes in the Retina of PD

Impairment of several specific physiological visual abilities in PD patients suggests that the retina may have suffered damage; therefore, it is of considerable interest to use techniques that can allow us to analyze this nervous tissue in a non-invasive way. The suggestion for this approach came from postmortem clinical studies highlighting that photoreceptors and RGC cells were swollen in PD patients [84].

Optical coherence tomography is currently the non-invasive technique for probing the retinal structure. With this technique, it is possible to estimate the integrity of the retinal ganglion cell nerve fibers by evaluating the thickness of the peripapillary RNFL in a reproducible manner [252,253,254].

With the OCT technique, thinning of retinal nerve fibers has been found in patients with Parkinson’s disease [71,72,73].

According to a previous report, OCT analysis revealed that the retinas of approximately 500,000 PD patients showed thinning of the macular ganglion cell–inner plexiform layer and retinal nerve layer [32].

In this era of innovation, the annotation workload for the experts in this field to identify PD signatures could be relieved using artificial intelligence (AI). This is exemplified by a recent study by Zhou et al., who organized an AI task to predict the incidence of PD based on retinal OCT images [255].

Another approach to investigate retinal differences in PD patients is to analyze ERG and VEP. These techniques are based on the principle that following a visual stimulus, electrical signals are generated in the eye and will be transmitted to the primary visual cortex. Therefore, to evaluate potential retinal damage in Parkinson’s patients, measurements of the amplitude and latency of these electrical responses can be compared with healthy patients.

These clinical approaches reveal distinctive features in PD patients, particularly a delay in the latency of VEPs [110], which is reversible upon administration of L-DOPA [109].

Similarly, ERG studies have shown alterations in both latencies and amplitudes in Parkinson’s disease [100,101,105], and these signals respond to the administration of L-DOPA [102,103,104].

## 5. Conclusions

Why investigate the prodromal signs of neurodegenerative disease in the retina? Several prodromal symptoms of Parkinson’s and Alzheimer’s disease involve the visual system, like decreased contrast sensitivity, vision color recognition, retinal thickness reduction, and electrophysiological anomaly. One compelling reason for studying these symptoms is given by the fact that the alterations of sensory abilities in vision are more easily and promptly detectable by patients themselves compared with tissue damage in the CNS, enabling them to easily report to their healthcare provider. These symptoms can be easily recognized up to 10 years in a prodromal form and also in the olfactory system [187].

Studies discussed in this review suggest that prodromal signs of neurodegenerative diseases observable in the retina could potentially complement currently used biomarkers, such as plasma/blood biomarkers (e.g., p-tau181 and α-synuclein), for the early detection of neurodegenerative diseases. Both strategies are still under investigation and require further validation to accurately differentiate between preclinical and symptomatic disease stages [256,257,258].

Furthermore, the National Institute on Aging (NIA) and the Alzheimer’s Association (AA) recently proposed a new classification system for Alzheimer’s disease (AD) based on the presence of ATN biomarkers (amyloid, tau, and neurodegeneration). Some studies suggest that plasma ATN biomarker measurements may effectively distinguish between AD patients and healthy controls, particularly when using techniques such as Illness Management and Recovery (IMR) for plasma amyloid β and the Simoa immunoassay for p-tau18. However, these two techniques are not widely or commonly used in the healthcare community [259].

It should be noted that older people affected by Alzheimer’s disease frequently have other co-pathologies such as those resulting from vascular lesions. Therefore, it cannot be excluded that these co-pathologies may also cause damage to the retina [260,261]. From this perspective, therefore, it is always necessary to associate evidence obtained from the analysis of the eye with other specific clinical and biomolecular investigations.

In this context, technological innovation in ophthalmologic tests will play a pivotal role in achieving more accurate and reliable results while reducing costs and time, thus enhancing clinical evaluation and providing specialists with the opportunity to practice personalized medicine. Therefore, these innovations may have the advantage of expanding screening and investigations for prodromal signs of neurodegeneration and assist in the diagnosis of co-pathological cases.

Among the most promising next-generation tools for detecting prodromal retinal alterations are new developments in optical coherence tomography: spectral-domain OCT (SD-OCT), which better delineates structural changes and fine lesions in retinal layers; swept-source optical coherence tomography (SS-OCT) and swept-source optical coherence tomography angiography (SS-OCTA), which give a better visualization of structure and vasculature below pigmented tissue with a larger field of view; and phase-variance optical coherence tomography (pvOCT), which can furnish detailed visualization of microvascular changes happening in the retinal tissue with 3D resolution [262,263,264].

Furthermore, enormous advances in image resolution arise from adaptive optics (AO)-based technology, which corrects for optical aberrations in the eye. This technique enables the collection of high-resolution images at the single-cell level of the human retina in vivo, with temporal and spatial optical resolution, significantly enhancing the study of eye structures in vivo [265,266].

The fluorescein angiography technique (FA) was widely used for the diagnosis of retinal and choroidal disorders prior to the introduction of OCT, which has emerged as a faster and safer procedure in routine clinical practice in recent years [267,268]. Noteworthy, this technique has recently shown improvement when coupled with adaptive optics scanning light ophthalmoscope (AOSLO) for the study of physiological and pathological vascular processes within the human retina in vivo [269].

In the future, as previously described for Parkinson’s disease, appropriately trained AI will be able to give more information on the probability of developing neurodegenerative pathologies with the investigation of retinal images [255,270,271].

Therefore, in light of a holistic approach to clinical findings, advancements in the development of novel biomarkers to predict neurodegeneration are still necessary, and retinal examination holds promise as a non-invasive, cost-effective, and easily accessible tool compared with neuroimaging of the central nervous system and cerebrospinal fluid analysis [256,257,258].

In Parkinson’s disease, symptom severity scores and the application of clinical-type biomarkers are currently the most reliable and validated methods for assessing disease progression, while laboratory-based biomarkers are mainly used during clinical trials and require further evaluation [272]. However, dietary factors, drug treatments, and sample preparation may interfere with clinical laboratory analyses, potentially leading to diagnostic errors [273]. A summary of the advantages and disadvantages of using the retina as a source of biomarkers compared with cerebrospinal fluid and blood is provided in Table 2.

Given this evidence, vision large-scale screening paired with appropriate sensitization and education of the population most susceptible to possible neurodegenerative problems could be desirable.

This article can inspire researchers and clinicians to look for new evidence of sensorial alterations that can be used to early detect and intervene before the onset of these most common neurodegenerative diseases in the worldwide population.

## Figures and Tables

**Table 1:** Visual-related dysfunctions in Parkinson’s disease and Alzheimer’s disease.

Ocular Manifestation	Parkinson’s	Alzheimer’s
Dopamine reduction	[44,45,46,47,48,49,50,51]	
α-synuclein accumulation	[52,53,54]	[55]
Amyloid β and p-tau protein accumulation		[56,57,58,59,60,61,62,63,64,65,66,67,68]
Retinal ganglion cell alteration		[65,69,70]
Retinal thickness reduction	[32,41,71,72,73]	[70,74,75,76,77,78,79,80,81,82,83]
Swollen neurons	[84]	
Visual acuity	[85,86]	[87]
Color vision	[88,89,90,91]	[43]
Contrast sensitivity	[51,88,92,93,94,95]	[96]
Motion perception	[97,98,99]	
Electroretinogram recordings	[100,101,102,103,104,105]	[106,107,108]
Visual evoked potential recordings	[109,110]	[106,107,111]

**Table 2:** Pros and cons of using the retina as a source of biomarkers compared with CSF and blood.

	AD-PD Biomarkers	Advantages	Disadvantages
**Retina**	OCT. ERG/VEP. Contrast sensitivity. Color vision. Visual field.	Established clinical tools. Cost-effective. Non-invasive. Accessible	Necessary association with clinical and biomolecular investigations. Co-pathologies may influence results.
**CSF**	Aβ. α-synuclein. p-tau. NfL.	High accuracy for diagnosis. Reflects real changes in the CNS. High concentration of the biomarker.	Invasive (lumbar puncture). Expensive. Time-consuming. Difficult to access. Withdraws small quantities.
**Blood**	Aβ. α-synuclein. p-tau. NfL.	Accessible. Non-invasive. Cost-effective. Easily measured. Large quantities available.	Lower concentrations of the biomarker are detected. Does not always reflect changes in the CNS. Dietary factors, drug treatments, and sample preparation may interfere.

## Data Availability

The data presented in this study are available upon request from the corresponding author.

## Abbreviations

AD, Alzheimer’s disease; APO-E, apolipoprotein E; APP, amyloid precursor protein; Aβ, amyloid beta; CatD, Cathepsin D; CNS, central nervous system; CSF, cerebrospinal fluid; DA, dopamine; ERG, electroretinograms; GCC, ganglion cell complex; GCL, ganglion cell layer; INL, inner nuclear layer; IPL, inner plexiform layer; MCI, mild cognitive impairment; NFTs, neurofibrillary tangles; OCT, optical coherence tomography; OPL, outer plexiform layer; p-tau, phosphorylated tau protein; PD, Parkinson’s disease; PET, positron emission tomography; RGC, retinal ganglion cell; RNFL, retinal nerve fiber layer; RPE, retinal pigment epithelium; SNpc, substantia nigra pars compacta; VEP, visual evoked potentials.
